# Can Clinical Outcomes Be Improved, and Inpatient Length of Stay Reduced for Adults With Diabetes? A Systematic Review

**DOI:** 10.3389/fcdhc.2022.883283

**Published:** 2022-05-18

**Authors:** Kathleen Michelle Friel, Claire McCauley, Maurice O’Kane, Michael McCann, Geraldine Delaney, Vivien Coates

**Affiliations:** ^1^ Department of Health and Life Sciences, Institute of Nursing and Health Research, Ulster University, Derry/Londonderry, Northern Ireland; ^2^ Clinical Chemical Laboratory, Altnagelvin Hospital, Western Health and Social Care Trust, Derry/Londonderry, Northern Ireland; ^3^ Department of Computing, Letterkenny Institute of Technology, Donegal, Ireland; ^4^ Altnagelvin Hospital, Western Health and Social Care Trust, Derry/Londonderry, Northern Ireland

**Keywords:** diabetes mellitus, length of stay, inpatient, clinical practice, clinical outcomes

## Abstract

**Aim:**

To examine the efficacy of clinical practice strategies in improving clinical outcomes and reducing length of hospital stay for inpatients with Type 1 and Type 2 diabetes.

**Background:**

People living with diabetes are at increased risk of being admitted to hospital and to stay in hospital longer than those who do not have the condition. Diabetes and its complications cause substantial economic loss to those living with the condition, their families, to health systems and national economies through direct medical costs and loss of work and wages. Length of stay is a major factor driving up hospitalisation costs relating to those with Type 1 and Type 2 diabetes with suboptimal blood glucose management, hypoglycaemia, hyperglycaemia, and co-morbidities shown to considerably impact upon length of stay. The identification of attainable evidence-based clinical practice strategies is necessary to inform the knowledge base and identify service improvement opportunities that could lead to improved clinical outcomes for these patients.

**Study Design:**

A systematic review and narrative synthesis.

**Methods:**

A systematic search of CINAHL, Medline Ovid, and Web of Science databases was carried out to identify research papers reporting on interventions that have reduced length of hospital stay for inpatients living with diabetes for the period 2010–2021. Selected papers were reviewed, and relevant data extracted by three authors. Eighteen empirical studies were included.

**Results:**

Eighteen studies spanned the themes of clinical management innovations, clinical education programmes, multidisciplinary collaborative care and technology facilitated monitoring. The studies demonstrated improvements in healthcare outcomes such as glycaemic control, greater confidence with insulin administration and reduced occurrences of hypoglycaemia and hyperglycaemia and decreased length of hospital stay and healthcare costs.

**Conclusions:**

The clinical practice strategies identified in this review contribute to the evidence base for inpatient care and treatment outcomes. The implementation of evidence-based research can improve clinical practice and show that appropriate management can enhance clinical outcomes for the inpatient with diabetes, potentially leading to reductions in length of stay. Investment in and commissioning of practices that have the potential to afford clinical benefits and reduce length of hospital stay could influence the future of diabetes care.

**Systematic Review Registration:**

https://www.crd.york.ac.uk/prospero/display_record.php?RecordID=204825, identifier 204825.

## 1 Introduction

Research has repeatedly shown that people living with diabetes are at increased risk of being admitted to hospital and to stay in hospital longer than those who do not have the condition (1–6). These studies associate diabetes-related complications such as cardiovascular disease, hypoglycaemia, hyperglycaemia, hypertension, and renal insufficiency with an increased risk of prolonged hospitalisation in addition to health risk factors including age, obesity and sedentary lifestyles.

Approximately 422 million adults are currently living with diabetes mellitus worldwide, with occurrence increasing among all ages strongly associated with increasing trends in obesity, unhealthy diets, physical inactivity and socioeconomic disadvantage (7). Diabetes and its complications cause substantial economic loss to those living with the condition and their families, to health systems and national economies through direct medical costs and loss of work and wages (8). It is estimated that the number of people living with diabetes globally will increase to 642million by 2040 and that even if countries meet internationally set targets, the global economic burden from the condition will still increase by 88% (9). In 2019, total, world-wide diabetes-related health expenditure was estimated to be USD 760 billion in adults aged 20–79 years, with much of the spending among those aged 50–79 years (10). Diabetes accounts for up to £14billion of the NHS England and Wales healthcare budget (11) and up to 14% of the Irish health budget (12). In the United States, more resources were estimated to be spent on diabetes than any other condition with people diagnosed with diabetes, on average, having medical expenditures ∼2.3 times higher than expenditures in the absence of diabetes (13). With the global costs of diabetes set to almost double to USD 2.5trillion by 2030 this condition can be considered a global health threat (9).

Length of stay is a major factor driving up hospitalisation costs relating to those with Type 1 and Type 2 diabetes (14). Suboptimal blood glucose management, hypoglycaemia, hyperglycaemia and co-morbidities have been shown to significantly impact upon length of stay and mortality rates (14). Extremes in blood glucose can occur in hospital due to disrupted self-management patterns, nil-by-mouth requirements, delayed mealtimes, inappropriate timing of medications and when the body is under stress (15). Numerous obstacles challenge the achievement of glycaemic control and there is a need to better understand the factors that influence blood glucose regulation within hospital care (16). The National Diabetes Inpatient Audit England (17) highlights that the rates of life-threatening harms such as severe hypoglycaemic episodes in inpatients with Type 1 diabetes, hospital-acquired diabetic ketoacidosis (DKA) and hospital-acquired hyperosmolar hyperglycaemic states remain unchanged despite being preventable. While much has been reported on practices that can impact hospital outcomes and subsequently length of stay, more evidence is needed to indicate how the situation can be improved.

The provision of inpatient diabetes care includes diabetes self-management, promptly responding to DKA and other acute complications and preparing patients for clinical procedures and eventual hospital discharge facilitated by a proactive and knowledgeable workforce (18, 19). Clinical staff are working within current guidelines and hospital protocols, which outline treatment pathways for much of the decision making around monitoring and acting upon blood glucose levels that are out of target range. They take steps to reverse hypoglycaemia, the administration of oral and injectable therapies and liaise with, and make referrals to, other members of the clinical team throughout the patient’s hospitalisation. These approaches cannot be working optimally if length of stay is longer for those living with diabetes (1–6)The application of attainable evidence-based clinical practice strategies is necessary to inform the knowledge base and enable competent decision-making within an innovative clinical environment to enact appropriate care in practice.

Despite current protocols and guidelines, the extended length of stay for those with diabetes remains a serious problem, indicating the need for further evidence about what works in practice and offering opportunities to close the gaps between evidence and practice in diabetes care (20–25). This review aims to explore the efficacy of clinical practice strategies (such as the organisation of care, the inclusion of specialist practitioners in the team, the use of protocols or guidelines and technology) in improving clinical outcomes and reducing length of stay for inpatients with Type 1 and Type diabetes.

## 2 Methods

A review of the literature was undertaken using Preferred Reporting Items for Systematic Reviews and Meta-Analysis (PRISMA) guidelines (26). This review is registered with the International Prospective *Register* of *Systematic Reviews *(CRD42020204825) (27).

### 2.1 Search Strategy

Three electronic databases (CINAHL, Medline Ovid, Web of Science) were searched for studies published between January 2010 and November 2021 with Scopus used for citation chaining and hand search of Google scholar. The search criteria used MeSH terms such as hospitals, community; hospitals, general; hospitalisation; diabetes mellitus;, Type 1; Diabetes Mellitus, Type 2; length of stay; intervention; nursing intervention and the following keywords in isolation or combination: ‘hospital’; ‘inpatient’; ‘ward’; ‘acute setting’; ‘diabetic adult’; ‘adult with diabetes’; ‘adult living with diabetes’; ‘person living with diabetes’; ‘intervention’; ‘treatment’; ‘program’; ‘strategy’; ‘service’; ‘plan’; ‘best practice’; ‘evidence base’; ‘evidence-base’; ‘tool’; ‘trial’; ‘length of stay in hospital’; ‘length of hospital stay’; ‘length of inpatient stay’; ‘length of stay in acute ward’; ‘length of hospitalisation’.

### 2.2 Study Selection and Eligibility

The population, intervention, comparison, outcome (PICOS) (28) model was utilised as a search strategy tool. Records were selected based on study eligibility criteria, i.e., must be/have:

1. Population: Inpatient diagnosed with diabetes ≥18 years old;
2. Intervention: Reports an intervention designed to improve clinical outcomes and reduce length of hospital stay;
3. Comparison group: Pre/post intervention design;
4. Outcome: Reports improved clinical outcomes and reduced length of stay as outcome variables;
5. Publication between 2010-2021 in order to capture the most contemporary practice; 6. Reports and audits published in a peer reviewed academic journal; 7. Publication in the English language, due to lack of resources for translation; 8. No restrictions on country of origin. Non-empirical studies, grey literature and other literature that did not report on outcomes were excluded. The search period was limited to 11 years to capture the most up to date evidence in rapidly evolving health care systems.

### 2.3 Data Extraction and Synthesis

**Figure 1:**
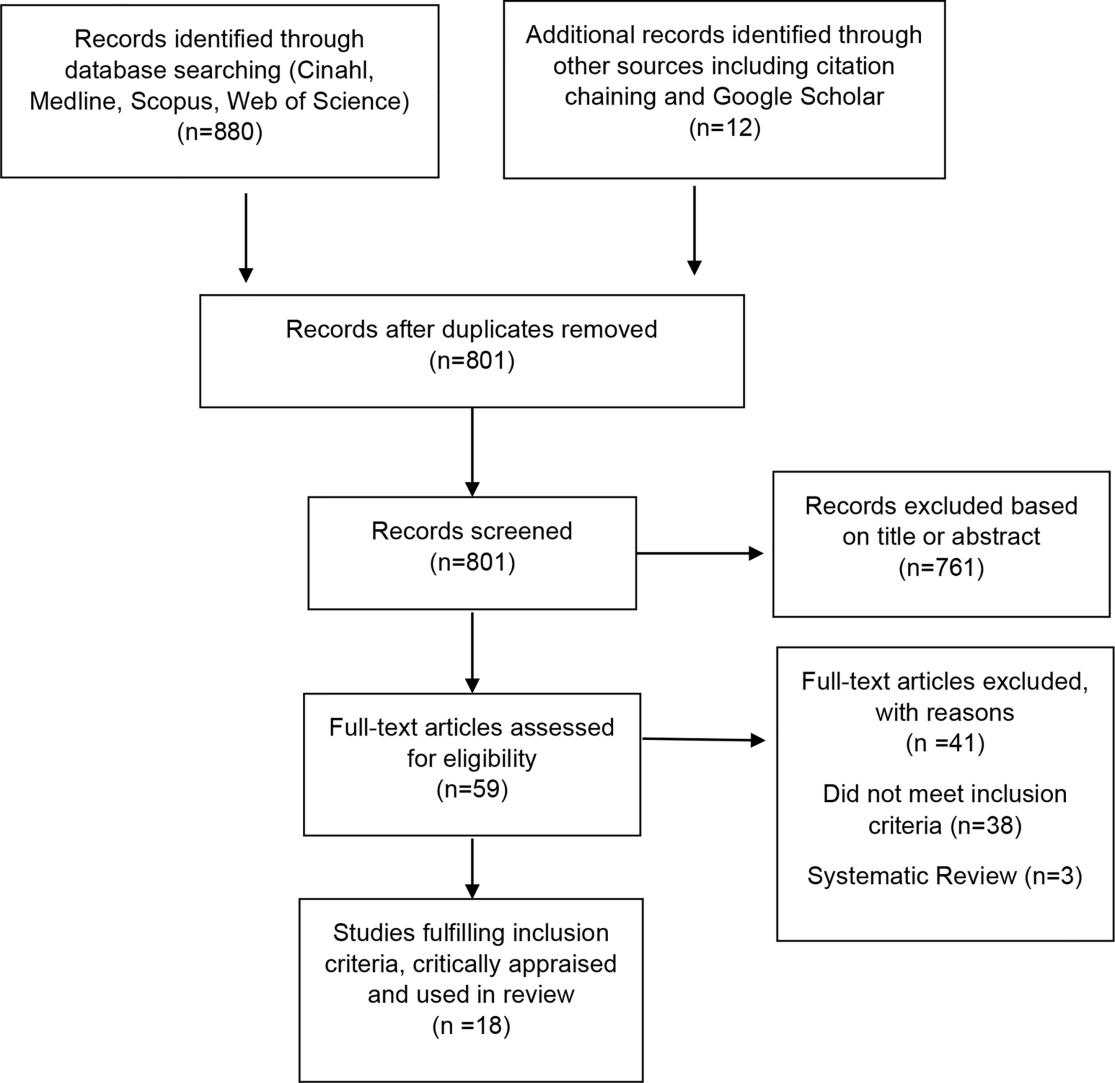
Flow diagram showing results of searches for systematic review.

Titles and abstracts were retrieved and screened for eligibility by one researcher [redacted] including 12 hand searched publications from citation chaining. Of those citations, 761 were excluded based on title or abstract and 59 full text articles were appraised. Eighteen studies were then reviewed, and relevant data extracted by three authors [redacted] individually. Any differences in opinion were resolved together through reviewer discussion. After consensus on eligibility was reached, a total of 18 studies were included (see **Figure 1**). The PRISMA checklist criteria (26) and the Cochrane Handbook for Systematic Reviews of Interventions (29) were applied. 

The data extracted included author, year, geographical location, design, gender and age, sample size, length of intervention, primary outcome measurement(s) and intervention results. The study designs and interventions varied, and outcomes were deemed too heterogeneous to perform a meta-analysis, therefore a narrative synthesis was undertaken together with an overview to summarise and explain the characteristics and findings of the included studies and of the extracted data.

## 3 Results

Eighteen studies were identified across 9 countries: UK n= 8 (30–37); USA n= 6 (32, 38–42); Israel n= 2 (43, 44); Canada n=2 (32, 45); Taiwan n=1 (46); China n=1 (47); Spain n=1 (32); Italy n=1 (32); and Ireland n=1 (32). The studies varied in terms of design; two randomised controlled trials (32, 46); six intervention studies (28, 29, 31–33, 38); eight retrospective, prospective audit studies (30, 31, 33, 41, 43–45, 47) and three service improvement studies (34, 37, 42). Whilst we recognise that service improvement and audits are not research, important information can be garnered from these studies where statistically controlled outcomes have been presented. All studies included adults living with diabetes that were ≥18 years old.

Investigations included adult patients (≥18 years) with Type 1 diabetes (n=17) (31–47) and Type 2 diabetes (n=15) (30, 33–38, 40–47). Inclusion criteria consisted of adults with a diagnosis of diabetes ≥18yrs, presenting to intensive care unit (ICU) (39, 43, 44), having a hospital stay of more than 48 hours (41), with diabetic foot ulcer, gangrene, cellulitis or infection as the primary cause of admission (31, 46), cardiovascular disease (47), diabetes ketoacidosis (38, 40, 45), hyperglycaemia (35), receiving insulin therapy (32, 42) and undergoing elective surgery (44). Research ethics approval and/or patient consent was obtained (32, 38, 39, 43, 45–47) with remaining studies regarded as audits, service, and quality improvement (30, 31, 33–36, 40–42, 44). Six studies (32, 34, 38, 43, 46, 47) used control groups and two studies (42, 45) described pre and post implementation groups. Clinical outcomes and length of hospital stay were comparably improved in studies using control groups compared with studies that did not include a control group. Four themes were identified: Clinical Management Innovations, Clinical Education Programmes, Multidisciplinary Collaborative Care and Technology Facilitated Monitoring. They are represented below for differentiation and readability purposes.

Methodological assessments were completed by two reviewers [redacted] with the Critical Appraisal Skills Programme (CASP) (48) used to assess the methodological bias of eighteen studies and provide the criterion for evaluating these papers. CASP (2019) (48) checklists were used in the appraisal of the studies to examine the research design, validity of the results, outcomes considered, recruitment acceptability and equality and studies providing sufficiently robust evidence that demonstrates improved clinical outcomes and reduced length of stay and where such evidence could be translated into clinical practice. The checklists identified that the studies included in this review had clear aims and results, met the desired eligibility criteria with low level of risk (*see* **Table 1** *Intervention studies with improved clinical outcomes and reduced length of hospital stay*). Limitations are most notable in the retrospective designs (30, 31, 33, 36, 41, 43–45, 47) small sample sizes (35, 36, 38–40, 42, 45) and single centre studies (30, 36, 38–40, 42, 44, 47) with novelty bias (31, 34, 36) and unequal allocation to groups (31, 34, 43). Three studies (34, 37, 42) utilised a quality, service improvement approach and although not research, we considered the outcomes to be significant for inclusion in this review. Fourteen studies presented statistically significant outcomes (30–33, 36, 38–40, 42–47), demonstrated adequately powered sample size (32, 39, 46) and made use of secondary data (30, 32, 35, 36, 40). Six studies were funded through non-commercial means (30, 32, 34, 36, 37, 47). This study does not indicate whether hospitalisations are diabetes related or non-diabetes related but examines conditions collectively as they relate to inpatient care. Strengths of the study include a comprehensive search of the major databases to identify all published empirical studies conducted on the topic. Furthermore, analyses of clinical interventions spanned a variety of practices that are already embedded in routine clinical care and/or require little change to implement.

**Table 1:** Intervention studies with improved clinical outcomes and reduced length of hospital stay.

Reference No.	First Author Year, Location and Study Design	Gender (%) and Age (Years, mean)	Sample Size	Length of Intervention	Primary Outcome Measurement(s)	Intervention Results
(30)	Akiboye et al., 2020 UK Retrospective analysis	2013: mean age ≈70.86; men ≈52.7% 2014: mean age ≈71.53; men ≈54.5%	n=7452 (2013) n=7635 (2014)	6 months	Length of stay and mortality	Reduced length of stay, increased mortality, no change in 30-day readmissions
(38)	Joyner Blair et al., 2018 USA Retrospective analysis	Men n=73	n=150	10 months	Treatment and management of DKA	Improved clinical outcomes, times for resolution of DKA and improved length of stay
		Female n=77				
		Mean age 43				
(31)	Chicero et al., 2013 UK Retrospective and evaluative	Age and gender not stated	n=109	21 months	Length of stay, rate of re-admission and difference in bed costs.	Improvements in length of stay, annual cost savings. No difference in re-admission rate.
		*Author contacted *via* ResearchGate and confirmed adult patients with Type 1 and Type 2 Diabetes.				
(46)	Chiu et al., 2011 Taiwan RCT	Intervention group: Men n=189 women n= 161 mean age 62.3yrs Control group: Men n=210 women n= 176 mean age 64.1	n=736	4 years	Clinical outcomes for patients infected with DFUs following a diabetic foot ulcer treatment programme.	Reduced length of stay, lowered amputation rate and faster time to complete recovery of blood glucose levels.
(32)	Feig et al., 2017 Ireland, Canada, England, Scotland, Spain, Italy and USA RCT	Women aged 18–40 years	n= 325	3 years	Change in HbA1c from randomisation to 34 weeks’ gestation in pregnant women and to 24 weeks or conception in women planning pregnancy, and was assessed in all randomised participants with baseline assessments	Intervention participants spent more time in target and less time hyperglycaemic.
						Neonatal health outcomes were significantly improved, with lower incidence of large for gestational age, fewer neonatal intensive care admissions lasting more than 24 h, fewer incidences of neonatal hypoglycaemia and 1-day shorter length of hospital stay.
(39)	Firestone et al., 2019 USA Retrospective study	Men ≈67%	n=201	4 years	HIIT and MIIT on clinical outcomes on the management of DKA and hyperosmolar and hypoglycaemic state	Reduced length of stay, prevalence and relative risk of hypoglycaemia lowered, and decreased glycaemic variability.
		Women ≈33%				
		Mean age ≈46.9%				
(33)	Flanagan et al., 2010 UK Retrospective study	Gender and age not stated	n=2287	1 year.	Length of stay.	Significant decrease in length of stay.
(35)	Herring et al., 2013 UK Interventional study	Adults aged 18 years or over	n=176	16 months	Length of stay and same-calendar-day discharges.	Increased same-calendar-day discharges, lowered length of stay, improved hyperglycaemia assessment, reduced use of intravenous insulin infusions and a high level of patient satisfaction.
(42)	Horton et al., 2015 USA Quality Improvement study	Mean age ≈53.9yrs Men n=≈53.3%, women n=≈55.4%	n=125	2 months	A change in the percentage of patients placed on basal-plus-bolus insulin regimens.	Decreased length of stay and in mean finger stick glucose.
(43)	Khalaila et al., 2011 Israel Prospective study	Intervention Group: Men n=46, Women n=48 Control Group: Men n=83, women n=54 Mean age ≈68	n=249	1 year	Conservative blood glucose control	Less occurrence of hypoglycaemic events and hyperglycaemia. Reduced length of stay.
(37)	Lawler et al., 2021 UK Service evaluation	Age, and gender not stated	n=7320 patient records	16mths	Impact of diabetes inpatient specialist nursing in 9 acute Trusts on length of stay and hospital readmission.	Decreased length of stay and reduced readmission rates.
		*Author contacted *via* email and confirmed adult patients with Type 1 and Type 2 Diabetes.				
(40)	Martin et al., 2016 USA Interventional study	At least 18 years of age	n=387	6 years.	Length of stay, hypoglycaemia and hypokalemia.	Decreased length of stay with no change in hypoglycaemia and hypokalemia.
		Gender not stated				
(45)	Mohamed et al., 2018 Canada Interventional study	Men n= 52% of the preintervention and 62% of the postintervention	n=110	3 years	Clinical outcomes from the implementation of a DKA protocol	Increased postprotocol ordering of appropriate laboratory investigations; improved appropriate intravenous (IV) fluid resuscitation; increased continuation of IV insulin until anion gap closure; decreased mean time to anion gap closure and mean length of stay was reduced. High levels of satisfaction from clinicians on improvements demonstrated by the protocol.
(34)	Page et al., 2017 UK Quality Improvement	N=53 participants in the passport Group; 42% women (mean age 69 years) 39 participants in control group 41% women (mean age 70 years)	n=92	Not stated	Improve the experience of perioperative care for people with diabetes and overcome some of the communication issues commonly identified in inpatient extracts	As a result of the passport, there was an increased prevalence of those who reported having received prior information about their expected diabetes care; the information given was significantly more helpful with passport participants more involved in planning their diabetes care, less anxious whilst in hospital and better prepared to manage their diabetes on discharge. Length of stay was shorter in the passport cohort, albeit not significantly.
(36)	Page et al., 2020 UK Interventional study	Baseline Group: Mean age 70yrs; 51% men Intervention Group: 71.3yrs; 60.2% men	n=351	1 year	Whether outcomes for people with diabetes undergoing elective surgery improve following the introduction of innovations in the peri-operative care pathway.	Significant decrease in mean HbA1c of those seen for optimizations by the diabetes peri-operative nurse. Recurrent hypoglycaemia significantly decreased and the mean number of hyperglycaemic events in people experiencing hyperglycaemia almost halved Mean length of hospital stay significantly decreased; 30-day readmissions did not increase. Postoperative complications significantly decreased.
(44)	Sharif et al., 2019 Israel Retrospective study	Mean age of 63.8; 73.6% men.	n=2466	3 years	To assess in-hospital glucose control on length of stay, 30-days and 1-year mortality.	Controlled glucose status was associated with shorter length of hospital stay; reduced 30-day mortality and improved 1-year mortality.
						Attainment of glucose control was independently associated with a significant decrease in 1-year mortality.
(41)	Sheahan et al., 2020 USA Retrospective study	Inpatients at least 18 years of age; men ≈55%, women ≈45%; age ≈55.93	n=11,477	5 years	To evaluate the impact of a diabetology consultation on length of hospital stay.	A diabetology consult within 48 hours of admission had a statistically significant shorter length of stay. No difference in complications or 30-day readmission rates.
(47)	Su XF et al., 2017 China Prospective study	Ward A n=944, 556 men, 388 women; age 66 ± 12	n=888	1.5 years	An intensive diabetes screening and treatment program to identify and treat diabetes mellitus in patients with cardiac disease	Significant reduction in medical costs and length of in-hospital days. Increased diagnosis rate of diabetes and impaired glucose regulation. More effective control of hyperglycaemia.
		Ward B n=554 347 men, 207 women; age 67 ± 10				

### 3.1 Intervention Classification and Outcomes

#### 3.1.1 Clinical Management Innovations (The Evidence)

Eight studies examined clinical procedures and treatments that lead to significantly improved clinical outcomes and reduced length of stay (34, 35, 38–40, 43, 45, 47). These included using moderate intensity insulin therapy versus high intensity insulin therapy for the management of diabetic ketoacidosis and hyperosmolar hyperglycaemic state (39). A significant reduction in the prevalence of hypoglycaemia (35% vs 1%; p=0.0003) and decrease in glycaemic variability by 28.6% (p<0.0001) were documented. Hospital and intensive care length of stay were significantly reduced by 23.6% (p=0.039) and 38% (p=0.017) respectively and the risk of remaining in hospital at day 7 (0.51; p=0.022) and day 14 (0.28; p=0.044). The evaluation of a clinical decision tool for non-diabetes specialists for the management of raised glucose (35) saw a reduction in the percentage of patients with hyperglycaemia as their primary reason for admission who then received an intravenous insulin infusion (84% to 56% (P<0.01). This resulted in a significantly lower median length of stay (3.5 days vs 1.0, P < 0.01). A high level of patient satisfaction was noted *via* a patient satisfaction questionnaire and estimated annual gross savings of more than £38K were also observed.

A protocol for the management of patients admitted for DKA was evaluated to determine if utilisation of the protocol versus an individualised provider approach for the treatment of DKA improved clinical outcomes (38). Results revealed decreased hypoglycaemia and improved insulin management with improvements in total mean length of stay (7.26 vs 5.21; p=0.1242) that were not significant. Similarly, a nurse-led intravenous insulin protocol was designed to achieve blood glucose control in patients using a format that allowed shifting between several algorithms corresponding to changes in capillary blood glucose values over time (43). The protocol showed improved glucose level time in target and hyperglycaemia and hypoglycaemia events occurring less in the intervention group with significantly shorter length of stay in the ICU (14.1 vs 11.6 days; p=0.04) and in the hospital (31.9 vs 27 days; p=0.03). High levels of satisfaction among nurses and physicians were achieved alongside standardisation of treatment, improved cooperation among interdisciplinary teams, greater confidence with insulin administration, and increased empowerment of the nursing staff (43).

A DKA critical care pathway was developed with the intent of standardising insulin, electrolytes, fluids, and monitoring with three key phases and requiring medical authorisation (40). The pathway significantly lowered length of stay with an average decrease of 104.3 to 72.9 hours (P =0.0003) after implementation with no change in secondary outcomes of hypoglycaemia and hypokalemia. Likewise, a protocol for DKA management was trialled with data abstracted concerning biochemical parameters, capillary blood glucose measurements, insulin and fluids ordered and received and precipitants of DKA (45). The protocol significantly increased the ordering of appropriate laboratory investigations from 60% pre-implementation to 91% (p<0.01) post implementation and improved appropriate intravenous fluid management from 33.3% to 93% (p<0.01) following protocol application. It also reduced mean length of stay (4.4 days vs 3.0 days) however, no level of statistical significance was reported. As a result of utilising the DKA protocol, 75% of patients rated the management of their DKA as satisfactory whereas nurses and doctors deemed the protocol to have improved patient care by 85% and 74%, respectively. The use of a perioperative passport was investigated to improve patient care and communication before and after surgery (34). Utilisation of the passport increased patient engagement in their hospitalisation and diabetes care resulting in positive feedback from patients regarding this perioperative tool and demonstrated a shorter mean length of stay (6.5 vs 4.4 days; p=0.059) although not significantly so (34). An intensive diabetes screening and treatment program for patients admitted with cardiac disease consisted of blood glucose control therapy, blood glucose monitoring and diabetes education (47). The programme, for patients with cardiac disease, especially ischemic heart disease, effectively controlled hyperglycaemia as both fasting blood glucose and two-hour post prandial glucose were significantly reduced (p<0.05) and significantly reduced length of stay (8.0 vs 7.0 days; p=0.002) in patients who received percutaneous coronary intervention. Medical and total in-hospital costs were reduced by 16.1%, with shortened pre- percutaneous coronary intervention hospitalisations time (5%) and total hospitalisation time (12.5%). Likewise, reductions in costs were also evident (22.8 and 21.8% respectively) in diabetes patients who did not require percutaneous coronary intervention (47).

#### 3.1.2 Clinical Education Programmes

The Diabetes Inpatient Care and Education programme demonstrated a unique approach by initiating an educational programme to inform staff about a pathway for patients with diabetes. The study incorporated an induction programme for junior doctors and employment of diabetes specialist nurses to facilitate the operation of the service (30). Patients without a diagnostic code of diabetes were used as a negative control group to assess the impact of temporal trends and changes in non-diabetes care processes that might impact on the outcomes of people with diabetes. Mortality rate decreased 6.4% to 4.4% with the adjusted odds ratio for the change in mortality pre- and post-intervention 0.63 (95% CI 0.48, 0.82) in people with diabetes. The programme revealed a significant reduction in lengths of stay for people with diabetes: relative ratios 0.89 (95% CI 0.83, 0.97) and 0.93 (95% CI 0.90, 0.96), respectively; however, in interrupted time series analysis the change in long-term trend for length of stay following the intervention was significant only for people with diabetes (P=0.017 vs P=0.48) (30).

A guideline-derived educational programme for resident doctors regarding inpatient glycaemic control and length of stay was analysed to encourage use of proper insulin regimens on inpatient glycaemic control and length of stay (42). Following the programme, a significant improvement was noted in the number of patients placed on basal-plus-bolus regimens (23% vs 8%; p=0.024) and length of stay significantly decreased (6.98 vs 5.03 days; p=0.042). Rates of hypoglycaemia significantly increased (4.6% -1.5%; p<0.001) whereas rates of severe hypoglycaemia did not (0.71% - 0.24%; p=0.089) (42).

Lawler et al. (37) presented a retrospective study examining the impact of diabetes inpatient specialist nursing across nine acute Healthcare Trusts that demonstrated varying decreased length of stay (significant in two Trust settings). This modelling paper involved data mining and while specifics were not detailed in which specialist nursing effected the change, it does allude to their role in education: ‘*The diabetes specialist nursing workforce plays a critical role in education of other healthcare professionals, and of patients, including promoting patient self-management’ (p.4085).*

#### 3.1.3 Multidisciplinary Collaborative Care

Six studies focused on multidisciplinary teams and diabetes specialist nurse positions (31, 33, 36, 37, 41, 46). A multidisciplinary Diabetic Foot Ulcer Treatment Programme consisted of endocrinologists, vascular and plastic surgeons in treatment that involved debridement within 12 hours, flap coverage and/or revascularisation to improve patient outcomes (46). The treatment yielded a lower amputation rate, a lower re-amputation rate and faster time to recovery of blood glucose levels. The programme showed a significant difference in hospital stay for patients with ischaemic infected wounds (33.8 ± 19.9 vs 24.5 ± 6.4 days; p=0.014) (46). The impact of a podiatric high-risk foot coordinator role was explored to facilitate more efficient and effective management of people with complex diabetic foot disease (31). Following the introduction of this position, extrapolated annual cost savings following implementation of this new position was £234K and the average length of stay was significantly reduced from 33.7 days to 23.3 days (p = 0.050). No significant difference in re-admission rates were observed (31).

A retrospective service evaluation examining the impact of diabetes inpatient specialist nursing across nine Healthcare Trusts utilised knowledge discovery through data mining from hospital episode statistics (n=7320 records) (37). Those Trusts that returned complete datasets (n=5) were found to have reduced readmission rates and varying decreased length of stay with two Trust areas showing significant decreases in length of stay and patient readmission after the introduction of diabetes inpatient specialist nursing. Improving the peri-operative pathway of people with diabetes used an approach for patients undergoing elective surgery and incorporated a diabetes peri-operative passport, diabetes peri-operative specialist nurse, surgical study days, a multidisciplinary group consisting of consultants, nurses, health care assistants and pharmacists, diabetes peri-operative champions and a new referral system (36). The peri-operative pathway significantly decreased postoperative complications (28% vs 16%; p=0.008) that included dysglycaemic complications, poor wound healing, wound and other infections (12% vs 5.4%; P=0.307) and mean length of stay (4.8 vs 3.3 days; p = 0.001). The mean glycated haemoglobin (HbA1c) of those seen by the diabetes peri-operative nurse significantly decreased, 84 mmol/mol (9.8%) vs 62 mmol/mol (7.8%; P ≤ 0.001), as did recurrent hypoglycaemia (7.0% vs 0.6%; P=0.002) whilst mean hyperglycaemic events almost halved (3.0 to 1.7; P =0.007) (36).

Flanagan et al. report the impact of introducing an inpatient diabetes team to improve healthcare outcomes and reduce length of stay. The team consisted of diabetes specialist nurses and a healthcare assistant that supported surgical, anaesthetic, and medical teams involved with the elective admission process. The work of the team concentrated on surgical care from pre-operation through to discharge (33). The team demonstrated a significant decrease in potential cost savings of £250K in bed days alone and in length of hospital stay of 0.34 days (P = 0.040). 93% of patients (sample size = 100) surveyed were happy with the advice given with 78% crediting the advice with improving their diabetes control in hospital (33). The impact of an inpatient diabetology consultation within 48 hours of admission on patients’ length of stay was evaluated (41). The consultation resulted in the probability of readmission decreasing following consultation within 48 hours of admission and a statistically significant shorter length of stay cut by 1.56 days (p<0.001) with length of stay reduced by nearly half a day for females compared to males (P=0.043) and evidence of this decreasing as individuals aged (P<0.001). Increased levels of illness and risk of mortality resulted in significantly longer length of stay. The probability of complications increased as a patient’s average point of care blood glucose value increased (P=0.028) (41).

#### 3.1.4 Technology Facilitated Monitoring

Two studies applied technology in the examination of improved clinical outcomes for patients admitted to hospital with diabetes (32, 44). The effectiveness of a continuous glucose monitoring device (iPro2 Professional) was examined on maternal glucose control and obstetric and neonatal health outcomes with patients wearing the sensor for 6 days and obtaining at least four capillary glucose tests daily (32). Continuous glucose monitoring device users spent more time in target (68% *vs* 61%; p=0.0034), less time hyperglycaemic (32% vs 27%, p=0.0279) with lowered time spent hypoglycaemic (4% *vs 3*%; p=0.10). Women with Type 1 diabetes randomised to continuous glucose monitoring during early pregnancy, had a small but significantly greater reduction in HbA1c levels (66%-52%; p= 0·0601). Neonatal health outcomes were significantly improved, with a 1-day shorter length of stay (p=0.0091), fewer neonatal intensive care admissions (0.48; 0.26 to 0.86; p=0.0157) and fewer incidences of neonatal hypoglycaemia (0.45; 0.22 to 0.89; p=0.0250) (32).

An in-hospital glucose control system was used to assess length of stay, 30-day and 1-year mortality with blood glucose measured by glucometer and input relayed to an interactive database (44). The system was associated with reduced 30-day mortality (4.6% vs 0.7%; p < 0.001), a significantly shorter length of stay (2.6 vs 1.6; p < 0.001), and improved 1-year mortality (7.5% vs 2.2%; p < 0.001) with glucose control associated with a significant decrease in 1-year mortality (p=0.047) (44).

## 4 Discussion

The findings in this review demonstrate significant improvements in clinical outcomes and in length of hospital stay across attainable clinical practice strategies. Multidisciplinary collaborative care exhibited the most impact with significant reductions in length of hospital stay utilising a podiatric foot coordinator position (31) and a foot ulcer treatment programme (46), revealing decreased length of stay by 10.4 days (31) and 6.4 days (46) respectively. A diabetes consultation with specialised diabetes teams within the first 48hrs was also effective with a decreased probability of readmission and reduced length of stay by 1.56 days (41). Clinical management innovations such as the introduction of a nurse-led implementation of an intravenous insulin protocol showed a significantly reduced length of stay (4.9 days) in addition to improved target glucose range and reduced hyperglycaemia and hypoglycaemia events (43). Furthermore, a clinical decision tool reduced length of stay by 2.5 days (35) and a DKA protocol decreased hospital stay by 2.05 days (38). In a guideline derived educational programme, while length of stay was significantly reduced (p=0.042), severe hypoglycaemia increased (0.71% vs 0.24%%; p=0.089), and rates of moderate hypoglycaemia (4.6% vs 1.5%) significant increased (42) thus demonstrating that the management of the patients’ condition worsened. However, the study does stress caution in any intervention that significantly increases rates of hypoglycaemia. With regards to technology facilitated monitoring, continuous glucose monitoring showed significant clinical improvements on maternal glucose control with less time spent hyperglycaemic (32% vs 27%, p=0.0279) and hypoglycaemic (4% vs 3%; p=0.10) with further clinical improvements exhibited for obstetric and neonatal health outcomes including reduced length of stay (p=0.0091) (32). A technology based in-hospital control of glucose parameters also indicated significantly shorter length of stay (2.6 vs 1.6; p < 0.001) (32, 44). These studies are important as they demonstrate significant improvements in clinical outcomes and reduced length of hospital stay. Yet the most recent NaDIA report (49) confirms that clinical inertia still prevails with 18% of hospitals reporting no dedicated diabetes inpatient specialist nurses, and access to diabetes specialist pharmacists and dietitians a continued problem, 18% of medication charts having one or more insulin errors during hospital stay and almost 1 in 3 inpatient charts having at least one medication error, unrecorded capillary blood glucose levels and 3.6% of inpatients with Type 1 diabetes having developed in-hospital DKA at any point during their hospital stay. So, there is an urgent need to implement evidenced based clinical practice strategies such as those outlined here.

Clinicians have key roles in multidisciplinary teams and notable responsibilities in clinical decision making in the management of blood glucose in consultation with other members of the clinical care team, all of which are shown to reduce length of stay. Organisational and individual initiatives are important to make evidence-based guidelines accessible with integration at ward level and individual practitioner level (50). With the use of clinical management innovations, multidisciplinary collaborative care, influencing pathways through education and technology facilitated monitoring, the programmes described here demonstrate reduced length of stay for the inpatient with diabetes. These innovations have a practical application in clinical care to enhance clinical, economic, and patient outcomes and address length of hospital stay. Particularly, the multidisciplinary collaborative care programmes described here consist of multifunctional teams (31, 36, 41, 46, 51) an approach that has been shown to support high quality and safe care, patient and staff satisfaction and engagement, and organisational efficiency and innovation (52) and which many healthcare systems now embrace (53). Interdisciplinary collaboration is also an important facilitation of communication between health professionals wherein medication errors are commonly affected by communication breakdowns (54).

Evidence from research must influence and shape healthcare professions, and inform and underpin policy, professional decision making and nursing actions to improve healthcare (55). The importance of enabling clinicians to develop evidence-based practice was recently articulated in the Chief Nursing Officer for England’s strategic plan for research (55) in which the ambition is to create a people-centred research environment that empowers nurses to lead, participate in and deliver research, where research is fully embedded in practice and professional decision-making, for public benefit. Supporting a practical research environment has relevance for health and social care providers and deans of university faculties engaged in healthcare education and research (56). The best studies could be taking place and the best evidence could be available, but if it is not assimilated into clinical practice and actioned, clinical inertia will prevail. Research active hospitals have been shown to have better patient care outcomes, contribute to a better inpatient experience and have a positive effect on staff and service performance (57, 58). An aging population, chronic disease management and costs of care are demands prompting healthcare reform. Technology can no longer be seen merely as an adjunct to practice (59) as healthcare professionals embrace technological changes such as digital communications, telehealth approaches and artificial intelligence in a bid to improve efficiencies and integrate modernisation. More studies are examining the use of technology in the optimisation of inpatient care and service delivery utilising this important tool for the improvement of healthcare quality and safety (60). Frameworks such as Promoting Action on Research Implementation in Health Services (56) can guide implementation, but clinicians cannot do this alone and cooperation is also needed from organisational management. As healthcare systems work under increasingly dynamic and resource-constrained conditions, evidence-based strategies are essential to ensure that research investments maximize healthcare value and improve public health (61).

This review examines interventions that demonstrate reduced length of hospital stay and significantly improved inpatient diabetes management. From the perspective of clinical practice, these are key considerations within the context of a dramatically changing clinical landscape. With diabetes resulting in extended length of stay for inpatients, differing clinical strategies and the integration of digital technology that effectively reduce length of stay are vital and could enable faster hospital discharge and lower healthcare expenditure. Results from these studies could minimise the burden the health service is presently experiencing by implementing evidence-based clinical practice strategies to improve patient outcomes, reduce length of hospital stay and efficiency of hospital care. Change is possible but requires the will and investment of health services and clinical leadership (58).

## 5 Conclusion

The clinical practice outcomes of this review contribute to the evidence base for inpatient care and treatment outcomes. The implementation of evidence-based research can improve clinical practice and show that appropriate management can enhance clinical outcomes and reduce hospital length of stay and improve hospital efficiencies for the inpatient with diabetes. With the rising prevalence of diabetes and the growing costs of the condition and its complications, adaptable healthcare is necessary for the future. Investment in and commissioning of practices that reduce length of stay, patient flow and hospital costs have the potential to afford clinical benefits and influence the future of diabetes care. As length of stay is a key performance indicator for the service, these results should be a catalyst for healthcare managers, professionals, and educators to facilitate the translation of such evidence into practice as well as to inform future policy.

## Data Availability Statement

The original contributions presented in the study are included in the article. Further inquiries can be directed to the corresponding author.

## Author Contributions

All authors listed have made a substantial, direct, and intellectual contribution to the work, and approved it for publication.

## Funding

This review is part of a wider study, the Centre for Personalised Medicine, Clinical Decision-Making and Patient Safety funded by INTERREG VA and managed by the Special European Union Programmes Body.

## Conflict of Interest

The authors declare that the research was conducted in the absence of any commercial or financial relationships that could be construed as a potential conflict of interest.

## Publisher’s Note

All claims expressed in this article are solely those of the authors and do not necessarily represent those of their affiliated organizations, or those of the publisher, the editors and the reviewers. Any product that may be evaluated in this article, or claim that may be made by its manufacturer, is not guaranteed or endorsed by the publisher.
